# Significant proportion of acute hepatitis B in Poland in 2010–2014 attributed to hospital transmission: combining surveillance and public registries data

**DOI:** 10.1186/s12879-018-3063-3

**Published:** 2018-04-10

**Authors:** Małgorzata Stępień, Karolina Zakrzewska, Magdalena Rosińska

**Affiliations:** Department of Epidemiology, National Institute of Public Health – National Institute of Hygiene, Chocimska 24, 00-791 Warsaw, Poland

**Keywords:** Hepatitis B, Epidemiology, Incidence, Surveillance, Vaccination, Modes of transmission

## Abstract

**Background:**

Efficient control of acute hepatitis B requires identification of current transmission routes. Countries in Central-Eastern Europe including Poland attribute an important fraction of cases to nosocomial transmission, as opposed to Western European countries. However, due to possible multiple exposures during the incubation time such assignment may be debatable. This study aimed at assessing of most affected groups and current transmission pattern of acute hepatitis B.

**Methods:**

We investigated exposures reported by acute hepatitis B cases notified to routine surveillance system in Poland in 2010–2014 in comparison to data on hospitalization rates in general population.

**Results:**

Hospitalization during incubation time significantly increased the risk of HBV infection (RR 3.13, 95%CI 2.58–3.80). Overall hospitalization population attributable risk (PAR%) was 25.7% (95% CI 20.3%–31.1%) as compared to 35% of acute cases assigned to hospital transmission in surveillance database.

PAR% increased from 9.5% (1.12%–17.8%) in the age group 25–34 to 41.1% (28.2% - 53.9%) among those 65 +. In addition, cases < 40 more frequently than the older ones reported history of injecting drugs and risky sexual contacts (25% vs 5%). 27% of men < 40 did not report any exposure at all, drawing attention to possible underreporting of risk behaviors.

**Conclusions:**

The distribution of probable transmission routes differed by age and gender. Further improvement of HBV control requires better coverage of vaccination in risk groups but also strengthening the blood-borne infections control in hospitals.

## Background

Hepatitis B continues to be a serious public health problem worldwide. According to the WHO estimates approximately 257 million people are infected with hepatitis B and over 880 thousands died in 2015 because of the late consequences of this infection [1].

Introduction of an effective vaccine against hepatitis B in the 1980s, significantly reduced incidence of this disease in many countries over the world. In Europe, depending on the epidemic situation, routine immunization of infants and/or targeted vaccination of risk groups was adopted [2].

Reported transmission routes differ between countries across EU. While over 40% overall, are attributed to sexual transmission [3], in some countries, especially in Central-Eastern Europe, considerable proportion of cases is thought to be due to nosocomial transmission. Whether or not it is really the case is debatable, due to long incubation period and possibly multiple exposures during this time. There is little information about possible molecular and/or environmental investigation of patient-to-patient transmission events from this region. In a review of published patient-to-patients transmission events in the EU and the USA, none of the analyzed events was reported from this region [4].

Poland was one of the countries with the highest hepatitis B incidence in Europe. The first noticeable decline was observed in 1986 attributed to the improvement of the medical equipment sterilization procedures, particularly gradual replacement of dry air sterilizers with autoclaves [5]. However, in early 1990s there were still 35 cases observed per 100,000 population, as compared to the European average of approximately19/100,000 at that time [5–7]. A real breakthrough occurred after the introduction of universal vaccination of newborns against hepatitis B in 1994–1996 [8, 9]. Later on, in 2000, a catch-up vaccination for adolescents aged 14 was introduced. In effect, the coverage among the birth cohorts born in 1986 or later as well in other groups targeted by immunization program, particularly health care workers exceeds 99% [9]. In adults born before 1986, vaccinated mostly in relation to planned surgeries or chronic diseases, the coverage is estimated at about 15–17% [8, 10, 11], although recent studies among attendees of primary health care suggest even higher values, exceeding 50% [10]. Nonetheless, acute hepatitis B continues to occur in the unvaccinated cohorts. The number of new acute infections depends also on the current prevalence of HBV infections which remain a source of new infections for the unvaccinated population. Prevalence of HBsAg reported in recent studies range from 0.9% to 1.12%. However, these data originate from studies conducted on subpopulations or small groups of people [12, 13]. An important source of information are data on HBV infections among blood donors - in the monitoring of blood donation within 20 years there was a decrease in the prevalence of HBsAg both among first-time (from 1% in 1994 to 0.3% in 2013) and repeat donors (0.1% to 0.02%) [14].

In addition to blood donors, all pregnant women, patients undergoing chronic dialysis, HIV-infected individuals, and persons applying for the refugee status are routinely screened for HBsAg. Existing care pathways allow to link all diagnosed individuals into appropriate care, including treatment with NA and PegIFNα2a.

Sustainable improvement of the epidemiological situation of hepatitis B depends on better understanding of the transmission patterns, both to target adult vaccination and to implement or improve other control measures. In surveillance data 35% of acute hepatitis B are attributed to hospital transmission and further 23% to medical procedures in the outpatient setting, although the evidence for such assignment is generally lacking (NIZP-PZH, unpublished data).

The aim of this study is to identify demographic groups in Poland who are the most affected with acute hepatitis B. Importantly, we compare the incidence among people, who were hospitalized during the past year and those who were not in order to quantify the role of potential transmission in the hospital settings. We use routinely collected data including infectious disease surveillance data as well as other public registries data, allowing to replicate the study over the time.

## Methods

### Data sources

The evaluation of the epidemiological situation was based on data on acute hepatitis B cases collected through the routine mandatory surveillance system in Poland between 2010 and 2014 (individual case reports). This period was selected to assure comparability of data, with respect to the case definition and the data sources. Over the analyzed period the system was comprehensive and based on reporting by clinicians. Reporting form for clinicians requires determining on what basis the disease was diagnosed (anti-HBc IgM, symptoms, epidemiological link or other). Cases reported as acute who did not meet the criteria required for confirmed or probable acute hepatitis B were qualified as unknown HBV (as to phase) and were not included in this study. Since April 2014, reporting of the positive test results by laboratories was introduced as parallel data source. In addition to the clinical report supplementary epidemiological data were collected (among other the history of exposures) by the local epidemiologists on a standardized form. Data on hospitalization derived from annual reports of National Healthcare Found publicly available from: http://www.nfz.gov.pl/zarzadzenia-prezesa/uchwaly-rady-nfz/uchwala-nr-42015ii,6343.html.

### Acute hepatitis B case definition

Cases of hepatitis B were reported to the State Sanitary Inspection by physicians who diagnose or suspect the disease on the basis of existing legislation. In 2010–2013, cases meeting the criteria of the case definition for acute hepatitis B approved by the EC in its decision of 2008/426/EC [15] for reporting communicable diseases to the community network were registered. According to this definition, acute cases were all symptomatic cases, laboratory confirmed by the presence of anti-HBc IgM and probable acute cases were symptomatic cases epidemiologically linked to a confirmed case of hepatitis B. In 2014 a new case definition for hepatitis B (2012/506/EU) was implemented with minor modifications to routine surveillance in Poland. In comparison to previous case definition, this introduced in 2014 excluded probable acute cases and laboratory criteria (positive results for specific anti-HBc IgM antibodies) were the only basis for the registration of acute hepatitis B case.

### Probable transmission route

The following exposures during 6 months prior to onset were ascertained through the reporting form: a) medical invasive and minimally invasive procedures by setting (hospital, outpatients clinic or nursing home), b) non-medical including: drug use (especially injecting use), home contact with HBV infected person, sexual contact with HBV infected person, sexual contact with people who inject drugs (PWID) or men who have sex with men (MSM) or multiple partners, tattooing, piercing, acupuncture, beauty treatments with skin puncturing, birth by a mother HBV infected during pregnancy, c) occupational exposure in medical or non-medical professions. Based on reported exposures probable transmission route was specified: medical procedures, PWID, sexual transmission including contact with HBV-infected and high risk sexual contacts, household contact with HBV-infected person, tattooing / piercing / beauty treatments, others (including occupational infections) or unknown/no data. In cases who reported more than one exposure the route of infection was classified into the above categories on the basis of the following hierarchy of risk factors: PWID, MSM, occupational exposures, chronic dialysis, heterosexual contact with a person infected with HBV, home contact with a person infected with HBV, > 1 sexual partner (heterosexual) during past 6 months and other including tattoo, piercing and beauty treatments. Since the risks associated with performing individual medical procedures are not well defined, in the case of multiple exposure medical procedures or hospital stay were set at the end of the list. The applied hierarchy was based on hierarchy proposed by Goldstein S [16] with modifications: we excluded blood transfusion from an infected donor, because after introduction of donor routine tests for HBV-DNA in 2005 risk associated with transfusions was reduced to a minimum [14, 17].

### Hospitalization

Hospitalization was defined as hospitalization due to any reason, but excluding nursing homes. For cases, the annual number of hospitalized in a given year was calculated as number of cases with the disease onset within this year reporting hospitalization during the presumed incubation period, i.e. within the 6 months prior to onset. For the general population we used a registered number of people who were hospitalized during a calendar year.

### Statistical analysis

Statistical analysis was restricted to cases in the unvaccinated birth cohorts (born before 1986). Average annual incidence was calculated using the reported cases and the available denominator estimates from the country statistics. Incidences by sex, hospitalization and employment status were compared using Student T-test or Chi-square test.

We calculated risk ratios (RRs) and 95% confidence intervals (CIs) for hospitalization, stratifying by the year of onset and age group. The population attributable risk (PAR%) and 95% confidence intervals for it was also calculated [18]. Chi-square test was applied for comparing category distribution, significance was defined as *p* value ≤0.05. Statistical analysis was performed using EpiInfo 3.5.3.

## Results

**Fig. 1:**
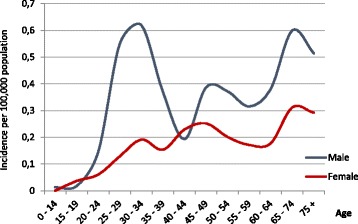
Acute hepatitis B in Poland in 2010–2014. Incidence per 100,000 population by age and gender

In total, 459 cases of acute hepatitis B were recorded within 5 years, including 19 cases from vaccinated birth cohorts who were discussed separately. The incidence in the analyzed period decreased from 0.34 per 100,000 in 2010 to 0.2 in 2014. Among all cases 65% were men, and 39% resided in larger cities (> 100,000 inhabitants). The incidence distribution by age was markedly different in men and women. It was increasing with age in women. In contrast, among men there was a distinct peak in the age group 25–34 (Fig. 1). Additionally, the incidence was higher among the unemployed than among working population (0.46 vs 0.25 per 100,000) and among those hospitalized in the past 12 months than among those who were not (0.71 vs 0.22). Table 1 presents the indicators in birth cohorts unvaccinated in infancy.

**Table 1:** Acute hepatitis B in Poland in 2010–2014 in birth cohorts unvaccinated in infancy. Distribution of reported cases and incidence by demographic features and history of recent hospitalization

		N	%	Incidence per 100,000	p-value^a^
Total		440	100	0.33	
Onset year	2010	123	28.0%	0.45	0.822
	2011	101	23.0%	0.37	
	2012	74	16.8%	0.28	
	2013	79	18.0%	0.30	
	2014	63	14.3%	0.24	
Sex	Male	285	64.8%	0.45	0.0011
	Female	155	35.2%	0.22	
Birth cohort-group^b^	1976–1985	133	30.2%	0.42	< 0.001
	1961–1975	96	21.8%	0.25	
	1946–1960	113	25.7%	0.28	
	born before 1946	98	22.3%	0.42	
Place of residence	Urban total	304	69.1%	0.35	0.0156^c^
	Cities< 100,000	129	29.3%	0.28	0.0149^d^
	Cities ≥100,000	175	39.8%	0.42	
	Rural	136	30.9%	0.26	
Professional activity (adults)	Employed	191	43.4%	0.26	< 0.001
	Unemployed	37	8.4%	0.58	
	Retirees	117	26.6%	0.37	
	Disability pensioners	42	9.5%	0.55	
Hospitalization history	Not hospitalized	274	62.3%	0.24	< 0.001
(during the incubation period)	Hospitalized	166	37.7%	0.74	

^a^Χ^2^ or Hartley’s f test

^b^Born after 1985 were vaccinated in infancy or at age 14

^c^For two groups: urban total vs rural

^d^For 3 groups: cities< 100,000; cities≥100,000; rural

The incidence rate among hospitalized was significantly higher for all the analyzed years, relative risk (RR) reaching the highest values in 2014 (RR = 4.20, 95% CI 2.56–6.88) (Table 2). The RR was significant for all age groups, lower for the age group 25–34, 1.86 (95% CI 1.19–2.91) as compared to the older age groups, among whom it ranged from 3.42 to 3.90. Overall population attributable risk (PAR%) was 25.7% (95% CI 20.3%–31.1%) and it increased from 9.5% (1.12%–17.8%) in the age group 25–34 to 41.1% (28.2% - 53.9%) among those 65 years old or older.

**Table 2:** Number of infected person hospitalized during 1–6 months before onset of symptoms, RR and PAR for this exposure

Year	N hospitalized^a^	N non-hospitalized^a^	Rate of hospitalization among infected	Rate of hospitalization in general population	Incidence among non-hospitalized^b^	Incidence among hospitalized^b^	RR	95% CI	PAR%	95% CI
2010	39	82	0.32	0.15	0.36	0.93	2.59	1.77–3.80	19.8%	10.0–29.7
2011	34	67	0.34	0.15	0.29	0.81	2.83	1.87–4.27	21.8%	10.9–32.6
2012	31	43	0.42	0.16	0.18	0.70	3.82	2.41–6.06	30.9%	17.6–44.3
2013	32	49	0.40	0.17	0.21	0.69	3.29	2.11–5.13	27.5%	14.7–40.3
2014	30	33	0.48	0.18	0.14	0.60	4.20	2.56–6.88	36.3%	21.3–51.3
2010–2014	166	274	0.38	0.16	0.24	0.74	3.13	2.58–3.80	25.7%	20.3–31.1
Age group										
25–34	24	95	0.21	0.12	0.34	0.62	1.86	1.19–2.91	9.5%	1.12–17.8
35–49	33	69	0.32	0.11	0.20	0.80	3.90	2.58–5.89	23.6%	13.6–33.6
50–64	44	63	0.41	0.16	0.18	0.67	3.65	2.48–5.36	30.0%	18.8–41.0
65+	65	47	0.58	0.29	0.24	0.82	3.42	2.35–4.97	41.1%	28.2–53.9
Total	166	274	0.38	0.16	0.24	0.74	3.13	2.58–3.80	25.7%	20.3–31.1

^a^ Only in groups unvaccinated in infancy or at age 14

^b^Per 100,000

People of other nationalities than Polish accounted for only 1.7% of patients and the imported infections constituted 3.3% of cases (15/459). Four imported cases concerned persons of a nationality other than Polish (Table 3). Most frequently reported exposures during incubation period were medical procedures (invasive or minor) (Table 3) - reported by 62% of all patients; 60% of cases were classified as transmitted by medical procedures based on the hierarchy. Medical procedures in hospitals were reported by 37% of cases and ultimately 35% of acute hepatitis B cases were attributed to hospital transmission.

**Table 3:** Acute hepatitis B in Poland 2010–2014. Distribution of cases by reported exposure and risk factors

		N of cases	%
Total		459	100.0
Exposure on hepatitis B risk factors	Any medical procedures	286	62.0
(more than one could be mentioned)	Medical procedures in hospital^a^	170	37.0
	Invasive procedures	96	20.9
	Medical procedures outside hospital^b^	114	24.8
	Tattooing/piercing or beauty treatments^c^	46	10.0
	Injection drug use	20	4.4
	HBV infected sexual partner or risky contacts	35	7.6
	HBV infected household	25	5.4
	Other^d^	12	2.6
	Unknown/none identified	97	21.1
Migration status	Polish origin	448	97.6
	immigrant	8	1.7
	unk	3	0.7
Possible importation	No	425	92.6
	Yes	15	3.3
	unk (transmission unknown)	19	4.1
Vaccination status	Fully vaccinated	29	6.3
	Incompletely vaccinated	27	5.9
	Not vaccinated	326	71.0
	Vaccination status unknown	77	16.8
Health care worker	No	456	99.3
	Yes	3	0.7

^a^Includes invasive and minimally invasive surgery, transfusion, dialysis, childbirths, biopsies, endoscopy, injections

^b^Includes dental treatments and extraction, procedures in outpatient clinics like injections, endoscopy, biopsy and other minimally invasive procedures

^c^Includes acupuncture, tattoo, piercing, botox cosmetic, mesotherapy, shaving

^d^Includes mother-to child-infections, occupational exposure, stay in prison, sniffing drugs, street fighting and accidental injuries

In most cases (315/459; 69%) infected persons reported only one known category of exposure within 1–6 months prior to the onset of disease, in 49/459 (11%) indicated two or more exposures of different categories, in 95/459 (21%) the route of infection was unknown.

Among 170/459 patients hospitalized in the incubation period 11 reported other exposure of higher risk of infection: 5 people among hospitalized injected drugs intravenously, 3 had risky sexual contacts and 3 - tattooing or beauty treatment.

**Fig. 2:**
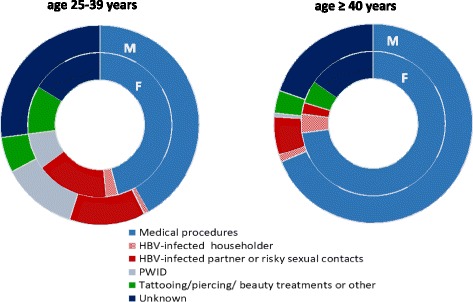
Acute hepatitis B in Poland 2010–2014. Distribution of cases by presumed route of transmission, gender and age

The distribution of presumed transmission routes differed by age among men and women. In those over 40 medical procedures account for over 70% of infections, while almost no infections are attributed to injecting drug use. In contrast, in the age group 25–39 sexual transmission and injecting drug use play important role assigned to 12.3% of cases in men in both categories and respectively16.2% and 8.1% in women (Fig. 2). Of note the proportion of cases with unknown transmission route was especially high among men in the younger age group (27%).

In total, 17 of 459 acute hepatitis B cases were positive for anti-HCV, including two coinfected both with HCV and HIV. In addition, one person was diagnosed with HBV, HIV and syphilis, with a negative HCV test result. Furthermore, in 3 cases positive for anti-HCV, HCV-RNA test results were negative, which indicates HCV infection in the past. For 32% of patients with acute hepatitis B, HCV status was unknown.

Among 18 people coinfected with HCV and/or HIV, 6 reported injecting drug use. Further 9 people with HBV/HCV coinfection were infected with HBV by medical procedures and in 3 cases the route of transmission was unknown (all possible exposures were negated).

HCV co-infections were significantly more common among HBV cases infected by drug use than among those infected another way (35% vs. 3.7%, *p* = 0.0001, in cases with complete information on transmission route and HCV testing). There were no HCV co-infections among people infected by sexual contacts, but there were 2 cases of syphilis.

### Acute hepatitis B cases in vaccinated birth cohorts

Of the 19 patients belonging to the vaccinated birth cohorts, 5 were classified as non-responders (fully vaccinated), 3 were vaccinated with 2 doses, 9 were unvaccinated (including 3 immigrants) and in 2 cases documentation was missing. Finally, there was one case of vertical infection that occurred despite prompt administration of HBIG and vaccine after birth.

## Discussion

We report HBV incidence in birth cohorts not covered by the routine vaccination program of new-borns in Poland, by demographic profile. The incidence remains significantly higher among men aged 25–39. Cases among men in this age group are more likely to report injecting drugs and sexual exposures (12.3% in each of these categories) as compared to cases amongst older men and amongst women. Further, combining surveillance data with the national health fund data, we identify an increased infection risk associated with hospitalization, suggesting possible transmission in the medical settings.

During 2010–2014, hospitalisation was associated with an over three-fold increase of the risk of acute hepatitis B (RR 3.13; 95% CI: 2.58–3.80). Based on the population attributable risk we conclude that 25.7% (95% CI 20.3–31.1) of cases may be attributed to exposures related to hospitalization, presumably because of poor adherence to universal precautions during medical procedures in hospitals. This is a remarkably high proportion in comparison to the countries in Western Europe and the USA, where the nosocomial transmission is reported in less than 10% of cases [3, 19] and often happen in community settings, nursing homes or dialysis units [4, 20–22]. It calls for revision of the infection control procedures with respect to blood-borne infections in medical settings.

Among cases who were hospitalized during the incubation period 54% had invasive (infection prone) procedures, i.e. major surgery or dialysis, and in almost half of hospitalized cases the stay in hospital was related to diagnostic procedures (endoscopy, biopsy, CT and MRI with contrast) or the conservative treatment with administration of drugs by injection or infusion. Such structure of exposures among cases may point to transmission during smaller procedures.

Moreover, for 18% (84/459) of acute hepatitis B cases, injections in an outpatient setting or outpatient dental treatment were listed as the only exposure during the period of 1–6 months before the onset of symptoms.

This is in line with other evidence of nosocomial transmission of blood-borne infections, including outbreaks reported from Poland and from other countries [4, 23–26]. The risk of infection associated with small procedures, unsafe injections and poor compliance with safety rules by medical personnel may partly explain the persistence of a large proportion of infections contracted in relation to medical procedures despite the large improvement in the conditions of equipment sterilization and success of the vaccination program. Among small medical procedures, attention should be drawn to the use of medications in multi-dose vials/packages or one vial of medication for more than one patient, inappropriate flushing of i.v. ports and cannulas, use of lancing devices, multiple use of disposable sets for contrast injectors for CT & MRI, and the insufficient knowledge and experience of personnel in this field [27–29].

Finally, the higher proportion of infections attributed to the health-care associated transmission should be also viewed in the context of background prevalence of chronic (undiagnosed) infection in the general population, following high incidence among young children in 1980s and 1990s.

The highest incidence was recorded among men aged 25–39 years (0.52 per 100,000). Moreover, in the age group 25–34 years PAR% associated with hospitalization was lower than in older age groups - 9.5% (95% CI 1.12–17.8). It may be associated with a shorter average length of stay in hospital of young people (5 days vs 10 days for persons > 65 years old and 8 days for all > 24 years old, but also higher prevalence of other exposures, e.g. injecting drugs or risky sexual contacts.

Within this group (men aged 25–39) the medical procedures represent 42% of the presumed transmission routes, compared to 60% in the whole infected population and 57% of all infected males. Infections among men aged 25–39 acquired by sexual contact (including MSM) and in relation to drug use represent in this group a higher percentage than among males ≥40 (25% vs 7%). Additionally, it is possible that the infections related to drug use or sexual transmission, especially among MSM are underreported. Unknown route of infection accounted for a higher proportion of cases among males aged 25–39 years than in all cases (27% vs 21%).

MSM and PWID are still disproportionately affected by HBV in Europe despite targeted vaccination campaigns in many countries [2, 30]. Immunization of these most at risk groups in Poland is not universal. The National Immunization Programme broadly recommends vaccination against hepatitis B for people at increased risk of HBV infection due to lifestyle or individual behaviors and the coverage in this group is likely similar to the general population. Previous research indicated that approximately 50% of PWID were immunized, but this proportion was below 30% among birth cohorts not covered by the routine immunization [31]. Targeting these groups would allow to further reduce hepatitis B incidence especially among younger adults. Men born in the 80’s and early 90’s should also be targeted for HBV screening due to the possible higher prevalence of chronic HBV infections in this group [6] - acquired perinatally or in early childhood in the period before the introduction of universal vaccinations of newborns. Currently, this is the main gap in testing policy in Poland – younger men, especially PWID (current and former) and with risky sexual behaviors, are not targeted effectively by testing practice.

### Limitations

Our study is limited by restricted availability of the population (denominator) data. As we were not able to individually link the surveillance and health fund databases we had to rely on aggregated data. The relative risk for hospitalization may be inaccurate since the recall time for cases was 6 months and the denominator included yearly number of people who were hospitalized. This is likely larger than the source population for our cases, thus we may obtain a conservative estimate of the relative risk.

Moreover, the true risk among exposed (hospitalized) may be higher also due to the fact, that part of the exposed population was actually vaccinated before planned admission and thus not susceptible to the infection. In a study among surgical/gynecological patients, over 60% were immunized and many of them specifically before the surgery [32]. Again this could bias the association of the recent hospitalization with hepatitis B infection towards the null. We therefore conclude that the risk exists and could only be higher that reported.

Another limitation is that we were not able to calculate the PARs adjusted for other exposures. Unfortunately, data on the population size of PWID and MSM were not available, especially if stratified by recent hospitalization. Finally we relied on the self-reported data. It cannot be excluded that some individuals did not report their sexual or drug injecting exposures. Thus these routes of transmission should be further investigated.

## Conclusions

In conclusion the ongoing HBV incidence may be attributed to two distinct transmission patterns: transmission in medical settings, including undetected outbreaks, and sexual transmission, especially among the men in addition to injecting drug use. Different measures may be appropriate to tackle HBV transmission in these groups, including vaccination campaign for MSM and PWID as well as strengthening the blood-borne infections universal precautions in hospitals. We were able to attribute a quarter of infections to hospitalizations and likely HBV transmission can also occur in the community settings. The ongoing health-care associated transmission indicates the need for continuous staff education and strengthening the monitoring of compliance with current guidelines, especially of injection safety.

## Abbreviations

CI: Confidence interval
CT&MRI: Computed tomography & magnetic resonance imaging
HBV: Hepatitis B virus
HCV: Hepatitis C virus
MSM: Men who have sex with men
MTCT: Mother-to-child transmission
NA: Nucleos(t)ide analogues
PAR: Population attributable risk
PWID: People who inject drugs
RR: Risk ratio, relative risk
